# Temperature Variability and Gastrointestinal Infections: A Review of Impacts and Future Perspectives

**DOI:** 10.3390/ijerph15040766

**Published:** 2018-04-16

**Authors:** Maryam Ghazani, Gerard FitzGerald, Wenbiao Hu, Ghasem (Sam) Toloo, Zhiwei Xu

**Affiliations:** 1 School of Public Health and Social Work, Queensland University of Technology, Victoria Park Rd, Kelvin Grove 4059, Queensland, Australia; gj.fitzgerald@qut.edu.au (G.F.); w2.hu@qut.edu.au (W.H.); sam.toloo@qut.edu.au (S.T.); z13.xu@qut.edu.au (Z.X.); 2 Institute of Health and Biomedical Innovation (IHBI), Queensland University of Technology, Musk Ave, Kelvin Grove 4059, Queensland, Australia

**Keywords:** gastrointestinal infection, diarrhea, climate change, temperature, heat wave

## Abstract

The objectives of this research are to review and assess the current state of knowledge of the association between environmental temperature and gastrointestinal (GI) infections. A review of the published literature was undertaken using standard approaches. Initially, four electronic databases including Embase, Medline, Scopus, and Web of Science were chosen to retrieve studies published from 1 January 2006 to 31 December 2017 based on selected keywords used in the primary search. After the elimination of duplicates, the titles were reviewed for relevance to the principal research question. Secondly, the abstracts of titles deemed to be relevant were reviewed for significance and finally the articles were reviewed in their entirety to identify their contribution to the principal research question. Initially, 8201 articles were identified, and eight studies finally met the inclusion criteria. A secondary phase involving scrutiny of the references of key identified articles found three further studies. Consequently, 11 papers were selected for the final review. Current literature confirms a significant association between temperature and infectious gastroenteritis worldwide. Also, a most-likely non-linear correlation between rainfall and GI infections has been identified in that the rate of such infections can be increased with either high or low precipitation. Finally, some studies suggest high relative humidity may not increase the rate of GI infections and some have found it may decrease it. These findings help inform predictions of risk, particularly under future climate change scenarios.

## 1. Introduction

For centuries, infectious diseases have caused serious challenges to human well-being and survival. One of the most common types of infectious diseases is gastrointestinal (GI) infection. Infectious GI disease accounts for a large proportion of mortality and morbidity worldwide [1]. Gastrointestinal infection (or infectious gastroenteritis) is a medical condition associated with inflammation of the gastrointestinal tract that involves both the stomach and the small intestine [2]. For instance, GI infection-induced diarrhea is known as the most common cause of death in the age group 0–5 globally [3].

Viruses, bacteria, and parasites are common causes of GI infections [4]. In most cases, the GI infection resolves spontaneously without any medical intervention; however, there have also been significant improvements in treatment options, which can reduce mortality and morbidity. The socio-economic impacts of GI diseases remain substantial, due to their high incidence [3]. Globally, infectious GI disease and dehydration remain leading causes of mortality, responsible for an estimated 450,000 annual deaths (8% of all deaths in 2016) [5]. The majority of the deaths occurred in children under 2 years old, and in South Asia and sub-Saharan Africa [5].

Thus, dehydration is less likely to cause death in developed countries, although gastroenteritis is a significant cause of morbidity. Annually, acute gastroenteritis accounts for 3.7 million physician visits, 135,000 to 220,000 pediatric admissions (9–13% of total hospitalizations for children below 5 years of age), and 150 to 300 deaths among children below 5 years of age in the United States (US) [2]. In Australia, it is estimated that there are 17 million GI infections per annum, nearly one episode per person [6].

The variable routes of transmission of these pathogens can be categorized into food-borne, water-borne, vector-borne, and person to person GI infections. The focus of this research is on food-borne disease because we hypothesize that there is an association between temperature and food-borne GI infections and that these may become more common under current climate change predictions. Food-borne GI infections may occur following food contamination during processing, transport, and preparation of food [7]. Some organisms causing food-borne infections, such as *Salmonella,* can survive in the general environment and replicate outside an animal host when conditions are optimal [7].

There is evidence that the growth and dissemination of the microorganisms can be influenced by weather, and some food-borne diseases show seasonality. For example, salmonellosis is most commonly reported in summer, whereas *Campylobacter* infections peak in spring. Reasons for the seasonal patterns could vary because the various pathogens have different characteristics, methods of replication, and methods of transmission. Seasonal temperature changes, which are usually associated with humidity and rainfalls changes, may also affect human behaviors around food consumption and social interaction. Akil and colleagues found that there is a strong positive association between prevalence of salmonellosis and increase in temperature [8]. There have been increasing concerns about the association between temperature variabilities and their impact on GI infections [9].

This research is conducted against a background of increasing societal awareness of climate change and its impact on human health. The changing climate is challenging human health in a number of ways, both direct and indirect, including changes in vector distribution, food supply, and disasters. Increases in global temperature, either in terms of longitudinal trends or as heat waves, have direct impacts on human health [10].

In prior studies, the possible influence of changes in ambient temperature as a climate change factor on the transmission of infectious diarrhea was reported [11]. For instance, there is a strong positive association between prevalence of salmonellosis and increase in temperature [8]. Other studies have identified the positive relationship between ambient temperature and pediatric GI infections [12]. However, the effect of climate variables, particularly temperature, on all age groups in different geographical areas and climate zones is not well documented. The aim of this article is to review the current state of knowledge of the association between environmental temperature and GI infections. Since temperature may impact the incidence of GI infections, in this research, the following questions have been examined: 1- What is the relationship between temperature and the incidence of food-borne GI infections? 2- How does extreme temperature, heat waves in particular, affect the rate of GI infections?

## 2. Materials and Methods

The aim of this article is to review the current state of knowledge of the association between environmental temperature and GI infections. The review was conducted in accordance with the Preferred Reporting Items for reviews and Meta-Analysis (PRISMA) approach. This literature review was reported by using the PRISMA checklist [13]. The study focused on reports of the impact on all causes of GI infections, so as to create an overview of the relationship between temperature and GI infections regardless of cause. The final articles were analyzed for consistent themes and these themes and observations were used to inform an understanding of the association.

## 3. Data Sources

Four electronic databases including Embase, Medline, Scopus, and Web of Science were searched for retrieve articles examining the association between environmental temperature and GI infections cases. Studies were included if they were published between 1 January 2006 and 31 December 2017 based on the United States National Library of Medicine (NLM)‘s Medical Subject Headings (MeSH terms) and using keywords including “gastrointestinal infection”, “diarrhoea”, “diarrhea”, “climate”, “weather”, “climate change”, “temperature”, “temperature variability”, “temperature change”, and “heat wave” (Table A1 in Appendix A). Additionally, the references of the included articles were manually checked to ensure all papers were included (snowballing).

## 4. Inclusion Criteria

Articles were included if they met the predetermined inclusion criteria as follows: 

(1) The studies addressed the effect of temperature (minimum, average, and/or maximum) or heat waves on the rate of GI infections. 

(2) The study included at least one continuous season of data to demonstrate seasonal fluctuation and to show the impact of temperature events on GI infections during a season.

(3) Full text was available, peer-reviewed, and published in English between 2006 and 2017.

Articles were excluded if they were not in peer-reviewed journals or an English translation was not available. Also, the studies that dealt only with a single GI infection pathogen were excluded. The reason was to have a better overall understanding of the effects of the climate variables on different pathogens.

## 5. Data Collection and Analysis

A total of 8201 potentially relevant articles were identified initially. After elimination of duplicates, the titles were reviewed for relevance to the research question. Secondly, the abstracts of those deemed relevant were reviewed for significance and finally the articles were 55reviewed in their entirety to identify their contribution to the research questions. As a result, eight studies finally met the specified inclusion criteria. A secondary phase involving scrutiny of the references of key identified articles found three further studies that had not been identified in the search strategy (manually checked).

**Figure 1:**
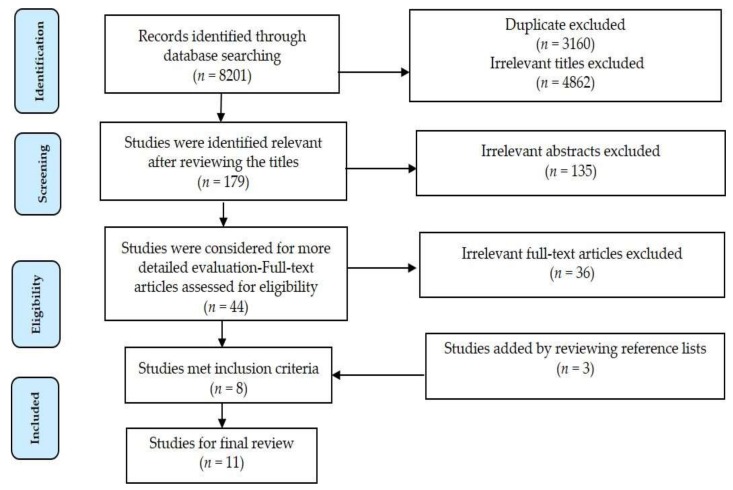
Literature search and study selection.

Consequently, 11 papers were selected for the final review. The eligibility of articles was assessed by two reviewers independently. A form was created for the extracted data, and the retrieved data included: publication details, location and time, unit of data, spatial scale, temperature, methodological characteristics such as research design and statistical analysis, outcome(s), key finding(s). Figure 1 shows the selection process and results.

The final articles selected for review were analyzed to recover their findings and to identify the extent of any association and any observable confounding influences. Because of the variability in definitions, methods, and outcomes it was not possible to undertake any meta-analysis of the data.

## 6. Results

The association between weather variables and GI infections was examined from a number of different countries with different environmental challenges including Scotland [14], Australia [15,16], the United States (US) [4], Switzerland [10], the Federated States of Micronesia [17], Japan [3,18], Vietnam [19], and China [20,21]. Table 1 presents the details of the identified studies, their characteristics, and key findings.

The studies reviewed demonstrate that a variety of methods have been used to analyze the association between temperature and GI infections. Studies varied in the metrics used to describe temperature (maximum, minimum, or average) while other papers examined the impacts of heat waves, which again are variably defined. They also described the impact of temperature and other climate variables and the nature of the variable associations with different causes of GI infection.

### 6.1. Statistical Methods

In order to show the association between weather factors and infectious gastroenteritis, a variety of statistical methods based on data availability were used by the above studies. Different types of Generalized Linear Models (GLMs) have been used. For example, a generalized linear Poisson model was adopted to examine the association between climate variables and climate-sensitive infectious diseases in the Asia-Pacific region [17] and Japan [3,18].

A Generalized Additive Model (GAM) was applied to evaluate the relationship between GI hospitalization numbers and weather parameters including temperature, humidity, and rainfall with 0–10 lag days [4]. To examine the lag period of weather’s impact on GI infection in Australia and Vietnam, a Distributed Lag Model (DLM) was applied [15,19].

A study into infectious gastroenteritis triggered by heat waves employed Poisson regression analysis to show the increase in hospital admissions [10]. Another study used a Quasi-Poisson regression model to assess the long-term and seasonal trend of physician-diagnosed GI infections associated to daily temperature [20].

### 6.2. Temperature and GI Infections

These studies demonstrate a variable association between temperature and GI infections. As temperature increases bacterial causes of GI infection appear to increase and this association is variably influenced by humidity and rainfall. On the other hand, as temperature decreases viral causes tend to increase and this does not appear to be impacted by humidity and rainfall. 

The associations have been explored in a number of settings and using different sources of data. For example, Phung et al. examined the correlation between temperature and hospitalization for GI and respiratory infections among young children in the Mekong Delta in Vietnam and found that the incidence of hospitalization increased with temperature above 24 °C and that such an association for GI infection was higher than for respiratory infection [19]. They also found that hospital admissions for GI infections increased during the wet season.

In a study in New York state, Lin et al. examined hospitalization for GI infections and found every 1 °C increase in temperature was correlated with a 0.70–0.96% increase in daily hospitalization for GI infections, particularly bacterial infections [4]. They also considered other meteorological factors and concluded that precipitation was significantly associated with increased hospitalization of GI infections [4].

Lam studied emergency department visits due to GI infections in children and concluded that every 1 °C increase in maximum temperature was associated with an 11% increase in the number of emergency department visits [16]. Lam also considered relative humidity and rainfall and found they were not significantly associated with emergency department visits for GI infections [16].

Two separate studies examined the association between GI infection and temperature in China. The first study (in Beijing) used mean, maximum, and minimum temperatures and found a high association between infectious diarrhea and temperature and also a high association with other environmental conditions such as relative humidity, mean water vapor pressure, daily precipitation, average wind speed, and sea level pressure [21]. The second study (in Shanghai) found that high temperature was a risk factor for infectious diarrhea disease among outpatient visits [20]. This study found no significant correlation with humidity and rainfall.

McIver et al. assessed the relationship between weather variables in the Federated States of Micronesia and found a significant association between temperature, rainfall, and GI infections [17]. An Australian survey found that community-acquired GI infections are substantially affected by weather. Every 1 °C increase in temperature is associated with an increase from the baseline of 2.48% and this association did not appear to be affected by rainfall and relative humidity [15]. 

The World Meteorological Organization classifies a heat wave as any 6-day period with maximum temperature >5 °C above the daily mean maximum temperature [10]. Manser and colleagues specifically considered the effect of heat waves on the incidence of infectious gastroenteritis in Zurich, Switzerland, between 2001 and 2005. They concluded that GI infections increased by 4.7% per every extra hot day during heat wave periods, resulting in increased hospitalizations [10]. Recent research by the same group has not demonstrated similar changes during cold snaps [22].

In Fukuoka, Japan, Onozuka and colleagues studied the impact of temperature on GI infections and found that every 1 °C increase in temperature was correlated with a 7.7% increase in the number of GI infections [3]. The study also found every 1% decrease in relative humidity correlated with a 2.3% increase in the number of GI infections [3]. In a further study, the researchers explored the association between temperature and childhood GI infections and demonstrated a 1 °C increase in temperature below 13 °C was correlated with a 23.2% increase in childhood GI infections, while every 1 °C increase in temperature above 13 °C correlated with an 11.8% decrease in childhood GI infections [18]. They also found that every 1% decrease of relative humidity was associated with a 3.9% increase of childhood GI infections [18]. These studies generally show an association between temperature and GI infections. 

Finally, in a study in Scotland, Eze et al. found that rising temperature increases the incidence of non-viral GI infections with an upward trend when peaks occur in July (summer), while decreasing temperature increases the incidence of viral GI infections in May (spring) [14]. They also found rising humidity increases the incidence of non-viral GI infections in July and decreasing humidity increases incidence of viral GI infections in May [14].

Although there are some inconsistencies in the results, the consistent message is that as temperature increases above baseline, bacterial GI infections will increase and this association appears to be enhanced by high humidity and possibly by rainfall. As temperature declines during colder months, then viral GI infections appear to increase, and this seems to be enhanced by lower levels of humidity.

### 6.3. Variability of Causes of Gastrointestinal Infections

The incidence of GI infections and the rate of outbreaks are seasonal and related to environmental parameters, particularly temperature [21]. There are different types of GI infection pathogens in different regions in the world and each type has its own habitat, characteristics, and ways of transmission [15]. In Scotland, Eze et al. found that the peak of GI infection was in summer (July), caused by non-viral microorganisms, particularly *Campylobacter* and *Salmonella* [14]. Onozuka and Hashizume, identified Norovirus and Rotavirus as the most common causes of GI infections in Fukuoka, Japan, in winter and spring, respectively [18]. In Australia, norovirus, rotavirus, *Giardia lamblia, Cryptosporidium*, *Escherichia coli (E. coli)*, *Campylobacter jejune*, and *Salmonella* are common causes of GI infections which have different responses to temperature [15]. For example, rotaviruses thrive in cooler conditions, while the rates of food-borne diseases due to *Salmonella* and *Campylobacter* have increased in summer and spring, respectively [15].

## 7. Discussion

There is international acceptance that the globe’s climate is changing as a consequence of anthropogenic activities, with current and projected changes increasingly becoming a serious threat for human health [23]. Surface temperature is projected to rise over the 21st century under all assessed emission scenarios. It is very likely that heat waves will occur more often and last longer, and that extreme precipitation events will become more intense and frequent in many regions. The ocean will continue to warm and acidify, and global mean sea level will continue to rise [24].

The frequency and intensity of natural disasters such as floods, droughts, bushfires, and cyclones are also increasing as the consequences of climate change [9]. Local changes of climate may be tolerated through behavioral, physiological, technological, and cultural adaptations; however, extreme temperature events may influence population health [25].

Climate variability is one of the most significant challenges facing humanity, and one which impacts on the environment, society, the economy, and human health and wellbeing [9]. It also has the potential to significantly affect human health in the future. It can impact on the host, agent, and environment (the classic epidemiologic triad), which are the factors by which climate can influence infectious diseases [26].

The modes of transmission of GI infections vary, and involve both environmental and host factors [3]. To have a better understanding of the potential impact of climate change on the total burden of GI infection, the effects of each climate variable on the infectivity of GI pathogen microorganisms should be estimated. However, this may be variable depending on the region and the pathogen profile.

Furthermore, children and adults should be studied separately, since each group has its own pathogenesis [15]. A popular opinion is that children, especially the school-aged group, are more susceptible to gastroenteritis as a consequence of temperature exposure, since children in this age group are more likely to be involved in out-door activities [16].

GI infections can be acquired via various exposure pathways such as person-to-person contact, food, water, and direct exposure to fecal wastes. As water consumption is higher during the hot seasons, this may increase the transmission of water-borne GI pathogens [20]. Any changes to climate variables may interfere with virulence, distribution, and survival of GI tract pathogens, jeopardize infrastructure, and vary host exposure patterns [27].

It is reported that both high and low rainfall may increase the rate of gastroenteritis with two different mechanisms. Heavy rainfall can contribute to contaminate water supplies with either human or animal fecal pathogens through flushing them from sewers and leaching them into water supply bodies (rivers and lakes). During low rainfall and drought periods, the concentration of fecal pathogens may increase. Therefore, when water scarcity occurs, there is increased use of untreated water with higher concentrations of pathogens [28].

Humidity is another climate factor which may impact on the incidence of GI infectious diseases. Some studies suggest the decrease in relative humidity is associated with significant increases in GI infection [3]. For instance, rotavirus replication and survival is enhanced through its increased aerial transport facilitated by low humidity [29].

This study reviewed the correlation between climate variables, temperature in particular, and GI infectious diseases. The significant effect of temperature on GI infections has been identified by previous studies, although the extent of temperature impact fluctuates in different regions. The review also showed that the contribution of temperature as an important climate contributor to GI infections is variable in different regions and with different infective agents. In areas with temperate climates, viral infective diarrhea peaks in particular seasons (rotavirus in spring and norovirus in winter) while in tropical/subtropical areas, they follow a year-round peak pattern [18,30].

There are significant limitations to obtaining a consistent and comprehensive understanding, identified in this study. The source of data can influence the observed association. For example, hospital admission data may be most diagnostically specific but reflects only a small proportion of cases; a proportion influenced by socio-economic and health system influences on hospital access. Weak clinical diagnosis and insufficient laboratory investigation has influenced the availability of incidence or prevalence data [3,16]. Among the 135 studies excluded, 6 articles were not available in full text. The relatively small number along with the lack of significance of the content meant that they would not have impacted on the overall findings.

There are other methodological issues that influence the observations. While population or community level weather parameters are identifiable, this does not necessarily express the population exposure. The latter is also influenced by socio-economic and behavioral factors including the nature and scope of community infrastructure.

This study has identified significant gaps in the understanding of the association between weather characteristics and GI infections. The majority of studies occurred in different regions and few have compared different climate sub-types [7]. Also, the impacts of climate factors on specific pathogens have not been examined sufficiently [3]. Moreover, there is not adequate knowledge of how extreme temperature events can affect pathogens transmission pathways. Transmission of GI pathogens is host related, and it seems that there have not been sufficient studies to raise public awareness and perceptions of heat impact on the GI health status of individuals [31].

## 8. Conclusions

Climate change is a worldwide problem and is leading to changing weather patterns and in particular to increasing temperature. The risk is that this will lead to higher rates of GI infection. The evidence is that there is an association between temperature and rates of GI infection. The increasing temperature and frequency and severity of heat waves due to climate change will increase the level of risk. The association varies with the type of organism: bacterial condition tends to increase with higher temperatures, while viral conditions may decrease as temperature decline. This association is affected by humidity and rainfall.

The effect of temperature on GI infections may be different between adults and children. As such, it is important to better understand the association to prepare models to identify the risk and to advocate for risk mitigation approaches.

This was an exploratory study and there are limitations to its scope. Only a small number of articles were found. We were not able to undertake a meta-analysis to demonstrate consistent associations because of differences in design, definitions, and outcome measures. There were significant differences in environment and context, which affects the generalizability of the results. Future research is needed to examine and compare the impact of temperature, age, and gender variables on the incidence of GI infections in different geographical areas so that targeted public policy solutions may be developed.

This paper reviewed the impact of temperature on the GI infections and identified future directions for research, focusing on the effect of temperature on GI infections, and informs the designs of early warning systems, which may help reduce the incidence of GI infections. A greater in-depth understanding of the association between climate and GI infections is needed in order to inform the development of risk mitigations strategies, such as health promotion and disease prevention. 

## Figures and Tables

**Table 1:** Characteristics of studies about weather variables and gastrointestinal (GI) infections.

Study	Location and Time	Unit of Data	Spatial Scale	Weather Variables	Research Design and Statistical Analysis	Outcome(s)	Key Finding(s)
[14]	West of Scotland 1998–2008	Monthly	2 Scottish National Health Service (NHS) board areas (Same coastal line)	Temperature Humidity	Time-series; Generalized additive models (GAM)	Incidence of GI illness	Rising temperature increases incidence of non-viral GI infections with peak in July. Decreasing temperature increases incidence of viral GI infections with peak in May. Rising humidity increases incidence of non-viral GI infections with peak in July. Decreasing humidity increases incidence of viral GI infections with peak in May.
[15]	Australia, 2001–2002 (Observation period)	Daily (Random selection)	Country	Temperature Rainfall Humidity	Time-series; Distributed Lag Model (DLM)	Community-acquired GI infections are substantially affected by weather.	Every 1 °C increase of temperature is associated with increase from the baseline (2.48%) of GI infections. Rainfall and humidity significantly correlated to GI infections.
[16]	Western region of Sydney, Australia, 2001–2002	Daily	City	Temperature Humidity Rainfall	Time-series; Autoregressive Integrated Moving Average (ARIMA)	Emergency department visits due to GI infections (children less than 6 years)	Every 1 °C increase in maximum temperature was associated with an 11% increase in the number of emergency department visits secondary to GI infections. Relative humidity and rainfall were not significantly associated with emergency department visits for GI infections
[4]	New York state, USA, 1991–2004	Daily	State level	Temperature Humidity Rainfall	Time-series; Generalized Additives Models (GAM) with a Poisson distribution and log link function	Hospitalization due to GI infection (long-term trend, seasonality, and calendar effects)	Every 1 °C increase in temperature is correlated to a 0.70–0.96% increase in daily hospitalization for GI infections, particularly bacterial infections, with lags from 1 to 4 days. Rainfall was significantly associated with hospitalization of GI infections, especially bacterial infections in a same lag period.
[10]	Zurich, Switzerland, 2001–2005	Daily	University Hospital of Zurich	Temperature	Retrospective controlled observational, Poisson regression	The effect of heat waves on incidence of infectious gastroenteritis	Hospitalizations due to GI infections increased during heat waves
[17]	The Federated States of Micronesia, Asia Pacific Region, 2000–2010	Daily	Country	Temperature Rainfall El Niño	Time-series; Generalized linear Poisson models	Relationship between weather variables and infectious diarrheal disease	Significant association were identified.
[3]	Fukuoka, Japan, 1999–2007	Weekly	City	Temperature	Time-series; Generalized linear Poisson models	Impacts of weather variability on GI infections	Every 1 °C increase in the average temperature was correlated to 7.7% increase in the weekly number of GI infections. Every 1% decrease in relative humidity was correlated to a 2.3% increase in the weekly number of GI infections.
[18]	Fukuoka, Japan, 2000–2008	Weekly	City	Temperature Humidity	Time-series; Generalized linear Poisson models	Hospital admission secondary to GI infections	Every 1 °C increase in temperature below 13 °C was correlated to a 23.2% increase in childhood GI infections among children under 15 years of age, while every 1 °C increase in temperature above 13 °C was correlated to an 11.8% decrease in childhood GI infections. Every 1% decrease of relative humidity was associated with 3.9% increase of childhood GI infections.
[19]	Mekong Delta, Vietnam, 2008–2011	Daily	City	Temperature Rainfall Humidity	Time-series; Poisson regression and constrained distributed lag model (DLM)	Hospitalization due to GI infections among young children	Incidence of hospitalization among 0–5-year-old children increases with rising temperature above 24 °C During the wet season with mean rainfall of 3.8 mm and mean humidity of 82%, hospital admissions increased
[21]	Beijing, China, 2004–2006	Daily	City	Temperature Humidity Water vapor pressure RainfallWind speed See level pressure	Epidemic risk level evaluation, Classical regression technique	Association between infectious diarrhea disease and meteorological factors	High association between infectious diarrhea disease and meteorological factors
[20]	Shanghai, China 2008–2010	Daily	City	Temperature Humidity Rainfall	Time-series; quasi-Poisson regression model	High temperature as a risk factor for infectious diarrhea disease in outpatients	High temperature is correlated to increased risk of GI infections among outpatients. Study did not find any correlation between increased risk of GI infections and rainfall. However, it found that humidity is associated to increased risk of GI infections among outpatients

## Appendix A

**Table A1:** Search strategy.

Database Name	Search Strategy
Embase	
Final S	“gastrointestinal infection” OR “diarrhea”/exp OR diarrhea OR “diarrhea”/exp OR diarrhoea OR “diarrhea/exp” AND (“temperature”/exp OR temperature OR “climate”/exp OR climate OR “weather”/exp OR weather OR “heatwave”)
Web of Science	
Final S	((“gastrointestinal infection” OR diarrhea OR diarrhoea) AND (climate change OR temperature OR climate OR weather OR “heat wave”))
Scopus	
Final S	(TITLE-ABS-KEY (“gastrointestinal infection” OR diarrhea OR diarrhoea) AND TITLE-ABS-KEY (temperature OR climate OR weather OR “heat wave” OR “climate change”))
Medline	
Final S	(“gastrointestinal infection” OR diarrhea OR diarrhoea) AND (temperature OR climate OR weather)
